# G23D: Online tool for mapping and visualization of genomic variants on 3D protein structures

**DOI:** 10.1186/s12864-016-3028-0

**Published:** 2016-08-26

**Authors:** Oz Solomon, Vered Kunik, Amos Simon, Nitzan Kol, Ortal Barel, Atar Lev, Ninette Amariglio, Raz Somech, Gidi Rechavi, Eran Eyal

**Affiliations:** 1 Cancer Research Center, Sheba Medical Center, Ramat-Gan, Israel; 2 The Mina and Everard Goodman Faculty of Life Sciences, Bar-Ilan University, Ramat-Gan, Israel; 3 Pediatric Immunology Service, Jeffrey Modell Foundation, Sheba Medical Center, Ramat-Gan, Israel; 4 Edmond and Lily Safra Children’s Hospital, Sheba Medical Center, Ramat-Gan, Israel; 5 Sackler School of Medicine, Tel Aviv University, Tel Aviv, Israel

**Keywords:** Variant, Mutation, Structure, Protein, Visualization

## Abstract

**Background:**

Evaluation of the possible implications of genomic variants is an increasingly important task in the current high throughput sequencing era. Structural information however is still not routinely exploited during this evaluation process. The main reasons can be attributed to the partial structural coverage of the human proteome and the lack of tools which conveniently convert genomic positions, which are the frequent output of genomic pipelines, to proteins and structure coordinates.

**Results:**

We present G23D, a tool for conversion of human genomic coordinates to protein coordinates and protein structures. G23D allows mapping of genomic positions/variants on evolutionary related (and not only identical) protein three dimensional (3D) structures as well as on theoretical models. By doing so it significantly extends the space of variants for which structural insight is feasible. To facilitate interpretation of the variant consequence, pathogenic variants, functional sites and polymorphism sites are displayed on protein sequence and structure diagrams alongside the input variants. G23D also provides modeling of the mutant structure, analysis of intra-protein contacts and instant access to functional predictions and predictions of thermo-stability changes. G23D is available at http://www.sheba-cancer.org.il/G23D.

**Conclusions:**

G23D extends the fraction of variants for which structural analysis is applicable and provides better and faster accessibility for structural data to biologists and geneticists who routinely work with genomic information.

## Background

Understanding the consequence of protein-coding point mutations is crucial to elucidate mechanisms of function and disease. This need has become even more urgent in recent years as next generation sequencing technologies and downstream pipelines typically identify many mutations which should be rapidly and accurately prioritized to assess their relevance. Most of the recent sequencing studies specify positions of interest according to the coordinates of the reference genome. Nevertheless, the numbering system of proteins in general and protein structures in particular, is by essence very different from that of the reference genome. Thus, to bridge the gap between genomic coordinates and those of proteomics and structural biology, efficient and convenient conversion tools are needed. To date, evaluation of genomic variants is mainly based on sequence features and sequence conservation scores. Structural data are rarely applied due to limited structural coverage of the human proteome and the above mentioned technical obstacles. However when applicable, structural data can be used to accurately calculate free energy changes, locate the spatial position of the residue with respect to known critical positions in the protein and known intermolecular interfaces. This issue is an example of the major challenge we are facing in the high throughput sequencing (HTS) era, which is to integrate efficiently and conveniently large amount of data from distinct origins. The tool presented here, G23D, enables mapping and visualization of genomic variants on three dimensional (3D) structures of proteins and helps integrate genomic data (either user provided or data from public databases) and protein structural data.

Tools for conversion from genomic coordinates to structural data already exist, but their functionality is often limited. MuPit [1] provides an interface to locate genomic positions onto available predetermined structures of the exact protein. Polyphen2 [2] and Mutation Assessor [3] provide structural information, albeit limited, for lists of variant. Some tools like SNPs3D [4], MutDB [5], LS-SNP/PDB [6] and Cn3D (http://www.ncbi.nlm.nih.gov/Structure/CN3D/cn3d.shtml) enable the user to explore variants which are stored at variant databases but not to upload new user defined variants. A number of other cancer related tools, including Cancer3D (http://www.cancer3d.org/) and the CBI portal (http://www.cbioportal.org/) also allow structural analyses. Tools which are not web-based exist as well [7, 8]. Ball-SNP [8] for example, allows integration of UCSF chimera with Cytoscape and other resources for integrative visualization of networks, sequences and structures. These tools enable the use of more sophisticated modelling features, but are obviously, less accessible to the community. There are also other good tools, like ELAPSIC [9] and SNPeffect [10] for analysis of functional, stability and protein-protein interactions changes upon point mutations which requires protein or structure information input, and do not support conversions between genomic and protein coordinates.

The new tool we present here, G23D, further helps to extend the utilization of structural data in several different ways. The structural data which is being scanned includes also homologous proteins and theoretical models. The importance of including homologous proteins in structural analysis has recently been demonstrated, in particular for analysis of interactions [11, 12]. G23D retrieves and displays hits from the PDB [13] which are not only identical but also similar to the input protein (i. e. homologous), under the well supported assumption that related sequences adopt a similar structural fold. Moreover, G23D also utilizes theoretical models from ModBase [14]. Thus, G23D facilitates structural analyses for a much broader space of proteins. We aimed to provide flexibility in selecting the appropriate structure template for a given genomic location. Therefore, the user can choose the most appropriate reference structure in each case and has considerable control over the filtering of the structural hits according to criteria such as similarity level with the input protein and the quality of the structure. G23D provides convenient and rapid conversion of genomic coordinates (or protein positions) to structural coordinates. It then allows instant on-line visualization of the structural context of the input variants, including modeling of the mutated amino acid and simultaneous visualization of the input variant alongside known pathogenic and non-pathogenic variants. Additional structure-based analysis features are provided as discussed below.

## Implementation

**Fig. 1:**
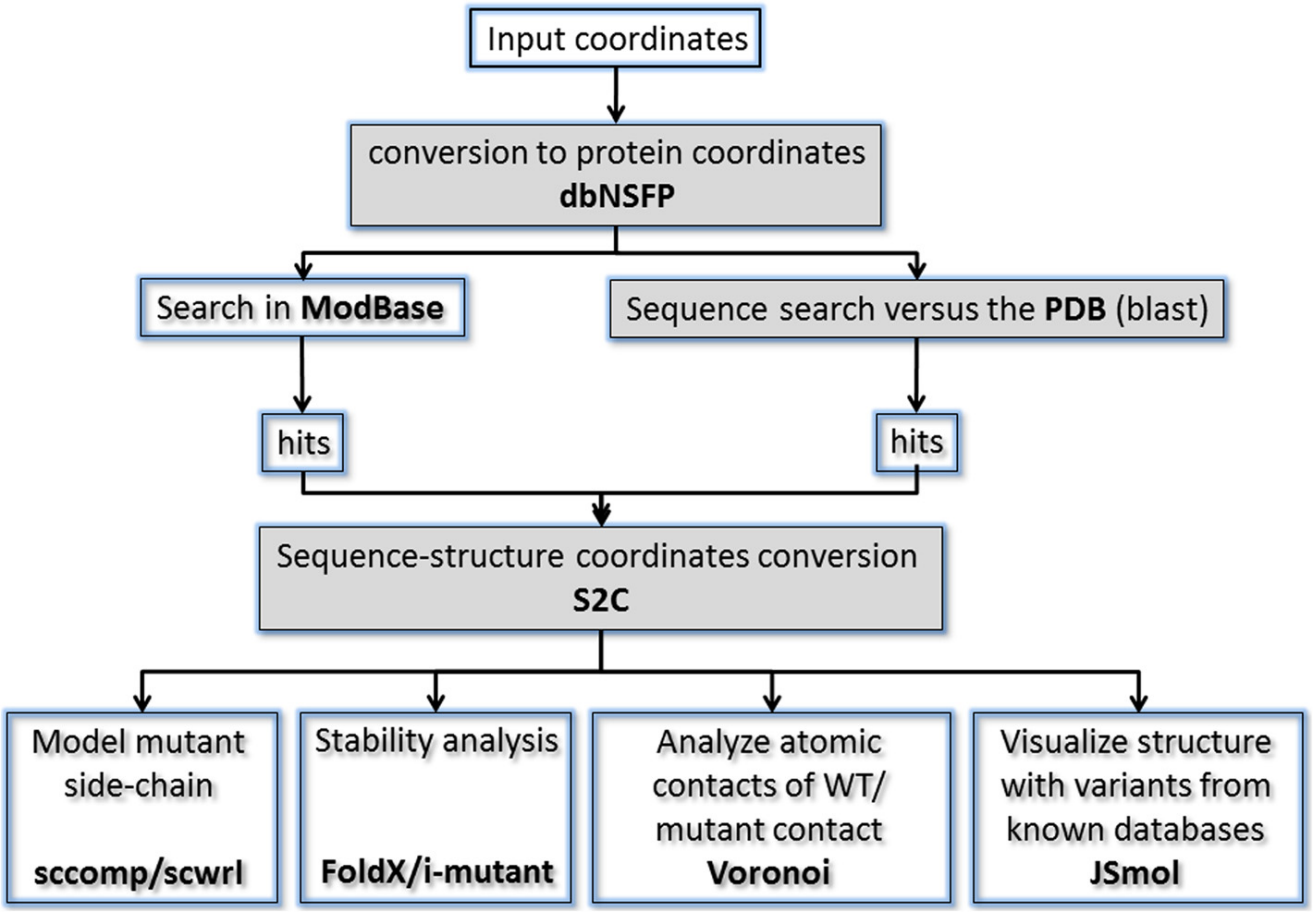
Schematic flowchart of the G23D pipeline and the resources being used. Genomic coordinates are converted to protein coordinates using dbNSFP. The proteins are then used to retrieve models from ModBase and their sequence is used for a blast search against the PDB. S2C (Roland Dunbrack lab) is used to convert sequence position to index of coordinates within PDB files. JSmol is used to visualize the hits. Side chain modeling programs are applied to model the mutant side chain. Comparative contact analyses using contact surface areas and stability analyses are based on the structure hits. Shaded boxes indicate steps which are pre-processed in advance and not during run-time

The most significant steps in the technical implementation of the site are the conversions between genomic coordinates and protein sequence coordinates and between protein sequence coordinates and protein structure coordinates (Fig. 1). G23D employs third party databases and newly developed offline pre-processing steps to obtain these conversions in advance.

dbNSFP [15] is used to convert genomic coordinates (GRCh37 or GRCh38 human reference) to uniprot protein coordinates. dbNSFP is frequently updated and currently version 3.1 is being used. Protein blast [16] is then applied for alignment of all Uniprot entries versus the PDB. The alignment not only retrieves PDB entries of the relevant proteins, but also detects structures of related proteins, either orthologs or paralogs, which can be used for structural analyses of the subject protein to a certain degree. S2C (http://dunbrack.fccc.edu/Guoli/s2c/) is applied for conversion of sequence and residue numbers from the PDB ATOM coordinates record.

The G23D site is mostly implemented in CGI combined with JavaScript and SVG. The 3D visualization is based on JSmol [17] which is a java-independent tool supported by all operating systems and major browsers without the need to install additional software or plugins. The site is composed of three main layers: an input layer, hits selection layer and an analysis layer. The requested variants are submitted to the input layer. The structures which cover these coordinates of the variants are then displayed in the hits layer, where hits can be filtered by various criteria. Selected hits can then be visualized and studied in the analysis layer which includes the JSmol molecular graphics session and additional structural information and analyses options. More detailed description of the various components is provided in the results section.

G23D employs data from many distinct sources. Proteins 3D structures are extracted from the PDB [13]. Protein theoretical models are retrieved from ModBase [14]. Protein sequences were downloaded from Uniprot [18]. Variants information was taken from COSMIC [19] and ClinVar [20]. Single nucleotide polymorphisms (SNPs) were taken from the common_no_known_medical_impact.vcf file of ClinVar (variants seen in healthy human population).

Protein residue conservation scores were taken from HSSP [21]. Motifs and domains were taken from Prosite [22]. Protein secondary structures and disordered regions were downloaded from DisProt [23].

As this work describes implementation of software and does not involve human materials, confidential human data or animal models, no ethics approval has been required.

## Results

### Description

**Fig. 2:**
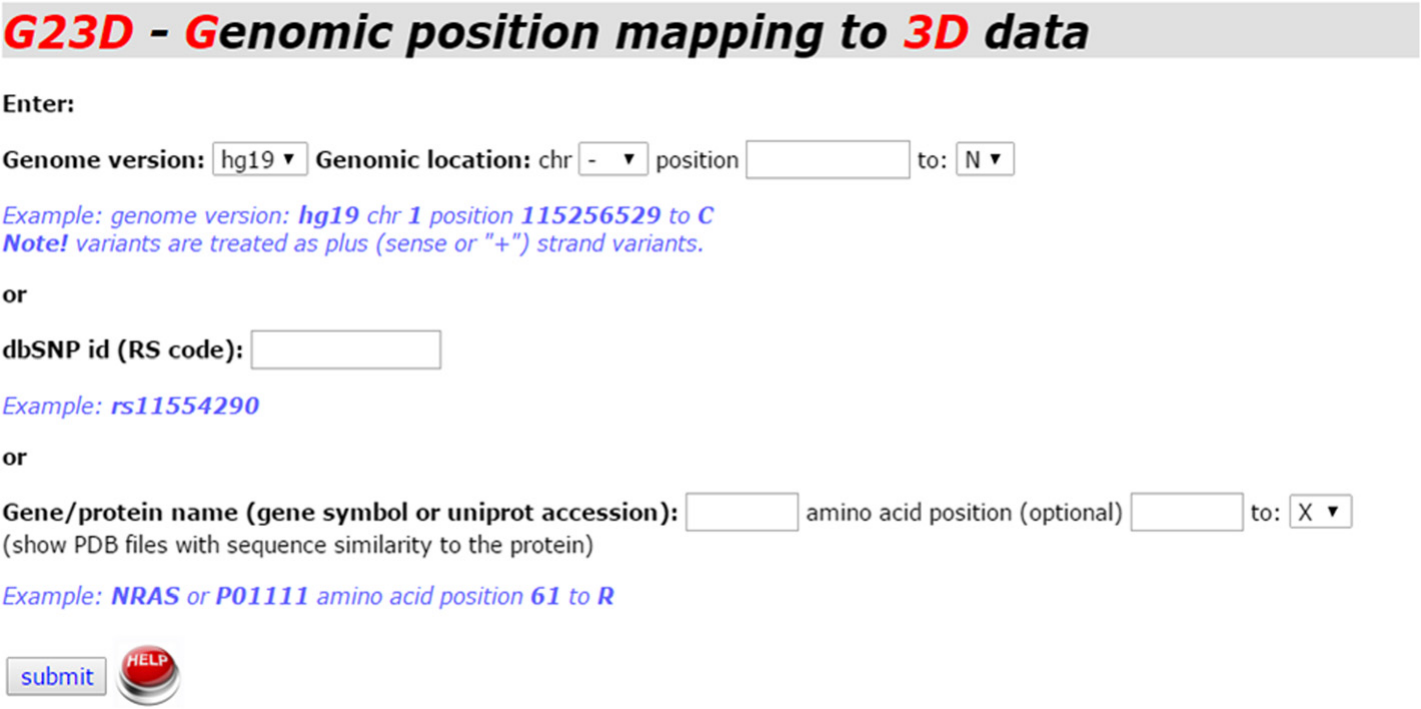
G23D front page includes the forms in which genomic coordinates, protein coordinates, dbSNP id, official gene symbol or protein name can be supplied. For genomic coordinates we support versions hg19 and hg38 (GRCh37 or GRCh38, respectively) of the human genome

**Fig. 3:**
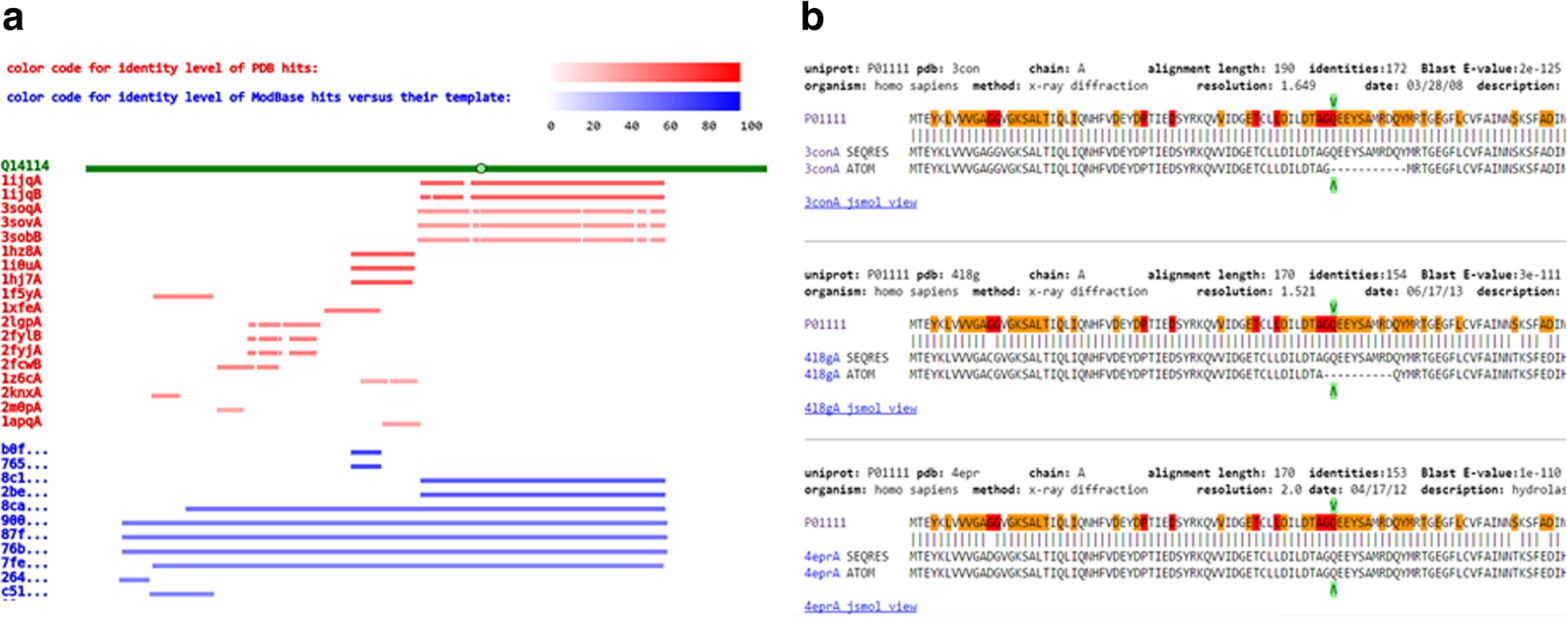
The hits’ page displays schematic and detailed alignments of the structural hits identified with the input protein (or the proteins) spanning the input genomic coordinates. **a** Schematic display of the alignment and the spanned regions. Horizontal colored bars, presented in a manner similar to Blast’s results page, serve as links to the detailed alignments located later in the page. The green bar indicates the query protein; red bars indicate PDB hits; while blue bars indicate ModBase models of the query proteins. Several isoforms might be found for the input protein. **b** Detailed alignments of the query protein (*upper sequence line*) and structure hits. Two sequence lines appear for the structure hit. One shows the protein sequence (PDB SEQRES record) and the second shows the sequence of the resolved coordinates (PDB ATOM record). In the first two alignments in this example (Q61 in NRAS) the loop which contains the variant is missing while in the third it is present. Description of the structure hits and indications for the alignment quality precede the alignment display. Residues which appear in dbSNP, Cosmic and Clinvar databases are indicated by various colors on the query sequence. The input variant is indicated by green arrows below and above the alignments. A link to the JSmol structure visualization page follows the alignment

G23D is a web-based tool which can be accessed by providing genomic coordinates, dbSNP id or protein position. Alternatively, an official gene symbol or a protein name can be provided as an input. Following submission of the requested information in the input page (Fig. 2) the coordinates are converted to protein space using dbNSFP [15]. A search is then performed to check which structure entries cover the input site. Sequences of PDB hits and ModBase hits are aligned to the sequence of the protein spanning the input coordinates. The user can easily explore the exact regions spanned by the structures and the similarity level to the protein of interest, as indicated by the color gradients (Fig. 3a). The same page also contains more detailed 3-way alignments of the structure hits (Fig. 3b). The first sequence is that of the query protein. Several query sequences may appear in this page as the mutation might be spanned by several distinct isoforms. The second line shows the sequence of the protein of the structure hit (SEQRES information in case of a PDB entry). The third line shows the part of the protein for which a 3D structure is available (ATOM part of the PDB entry). Missing domains, disordered regions and other undetermined regions will therefore not appear in this line. The amino-acid position affected by the input mutation (if provided) is highlighted. A link to the JSmol visualization page follows the alignment.

**Fig. 4:**
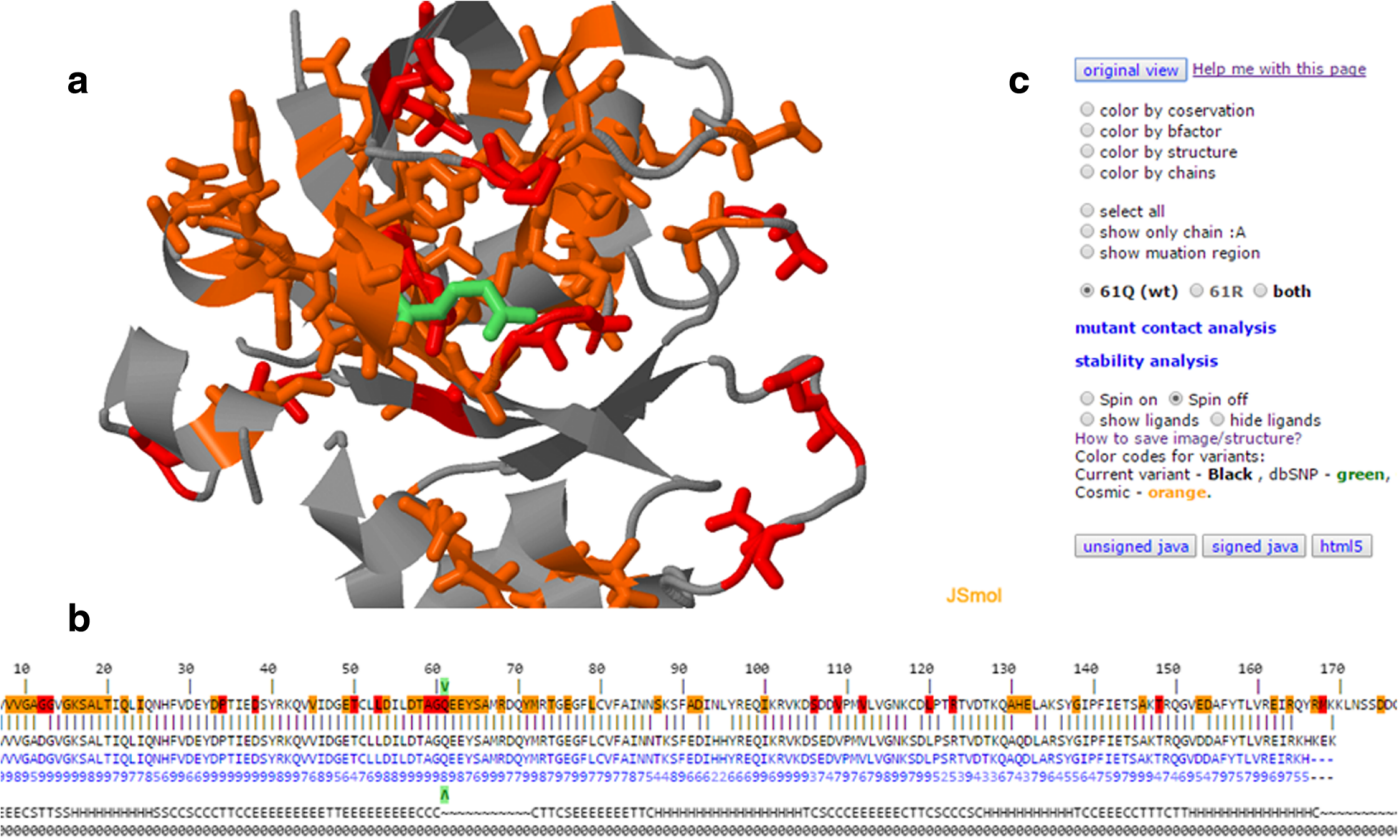
The structure visualization page is divided to three panels. **a** The protein is shown in cartoon representation, and the input variant (*light green*) and database variants (*red*, *yellow* and *green* with the same color scheme of the sequence alignment) are shown in stick representation. The sequence alignment **b** is similar to that found in the hits’ page. Additional layers of information such as conservation, secondary structure and disorder may follow the sequences. Residues on the sequence can be marked on the structure diagram. The Control panel **c** provides easy access to some common graphical features, alternate visualization of the wild-type and the mutant structures and links to contact analyses and stability predictions

The selected structure hits are displayed in a JSmol session (Fig. 4). The 3D display is shown in the center (Fig. 4a) in cartoon representation. The input variant, if indicated in the input page and included in the structure, appears in black. Database variants (if present) appear in the same color scheme of the sequence alignment and are shown in stick representation. The structural context of the input variant can therefore be evaluated with respect to known variants. Currently, G23D displays data from dbSNP [24], ClinVar [20], Cosmic variants [19] as well as catalytic residues from Catalytic Site Atlas [25]. Interactive sequence alignment, similar to that found in the hits’ page, is located in the bottom of the page (Fig. 4b). Residues selected in the sequence panel are highlighted on the structure. This panel includes also information regarding conservation, secondary structure and disorder regions, if available. Prosite [22] motifs are also shown in the sequence panel and are mapped to the structure. The Control panel (Fig. 4c) allows manipulation of the structure sessions (many more control options are available in the JSmol menu). The control panel also contains links to the contact analyses and stability predictions.

Using the combined sequence-structure presentation, the user can easily explore the structural context of selected sequence locations and patterns. The sequence conservation values from HSSP [21] can also be displayed on both the sequence and the structure using conservation color scale.

### Side chain modeling and contact analysis

**Fig. 5:**
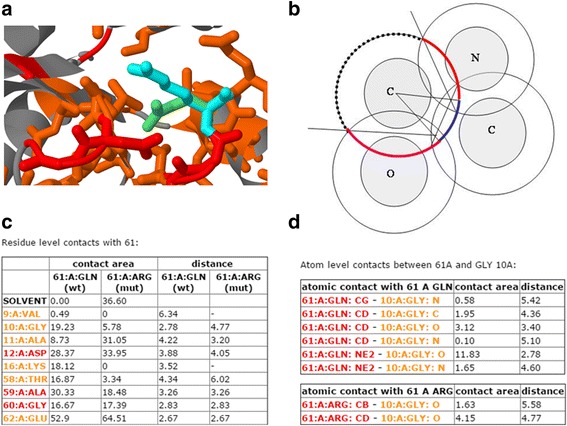
Modeling and stability prediction features in G23D. **a** A G23D model displaying the mutant structure instead, or together with, the wild type. The modeling is done using Sccomp, a side chain prediction program. **b** Contact surface areas between atoms and solvent accessible surfaces are analytically calculated using Voronoi tessellation. This procedure allocates the surface area of each atom to its neighbors (*colored arcs*) and the solvent (*dashed arc*). **c** A residue-level summary table lists all contacts and minimal distance to contacting residues for both wild type and mutant structures. Database residues are colored according to the general coloring of G23D. **d** By pressing on a residue in the left column of the residue-level table, the user can explore all atomic level interactions between the input variant and the chosen residue

G23D is not solely a visualization tool, as it provides additional modeling and analyses features which generally cannot be found in equivalent tools. The amino acid in the mutated position is modeled using SCcomp [26] or Scwrl [27]. Both programs are quite accurate but it should be stressed that both methods model side chains on a fixed backbone, so if backbone conformational changes are involved the model may not be accurate. Therefore caution is needed when the mutant side chain is larger than the wild type side chain in the protein core. In the 3D interactive session the user can explore the side chain conformation of the mutant, instead or alongside the wild type residue (Fig. 5a).

Given the structure of the wild-type protein and the mutation position, the user can explore the molecular contacts in which the residue participates. Contact surface areas and solvent accessible surface areas are calculated using analytic procedure, as described in Mcconkey et al. [28, 29], which applies Voronoi tessellation to allocate contact surfaces between neighboring atoms. The remaining surface of each atom, not assigned to contact with other atoms, is the solvent accessible surface (Fig. 5b).

The user obtains a table (Fig. 5c) which summarizes the data at the residue level. Each line in the table provides information on a single residue which forms contact/s with the residue coded by the input variant. These contacting residues are colored according to the same coloring schemes of G23D to help the user assess their significance. The information in the table includes the contact surface area (Å^2^) and the minimal atomic distance (Å) between any two atoms of the two residues. The user can further explore atomic contacts of contacting residues. Pressing the residue number in the left column of the table opens a new table, which provides atomic level information regarding interactions between atoms of the mutated residue and atoms of the interacting residue (Fig. 5d), including inter atomic contact surface area and inter atomic distance. Altogether, the user can evaluate the effect of the mutation by comparing the contacts and solvent accessible surface area of both the wild type residue and the mutant residue. The user can also manually examine the possible effect of the mutations over the 3D molecular graphics session.

G23D also provides links to third party programs which predict the consequences of the mutation by various considerations. If the variant is supplied by its genomic coordinates, then a link to the data of the relevant entry in dbNSFP [15] is provided. This database holds pre-compiled predictions for each protein coding variant in the genome regarding the possible functional significance of the variant. Predictions are available for eight different popular programs including SIFT, polyphen2, LRT, MutationTaster, MutationAssessor, FATHMM and PROVEAN. Evolutionary conservation is the most important individual feature in these predictions which generally do not consider structural features. Consensus predictions (i.e. meta predictions) are also provided and are in principal more accurate than the individual predictors [30].

In case structural information is available, G23D provides links to two different programs for thermostablity predictions, FoldX [31] and I-Mutant-2.0 [32]. The predictions of these tools are available from the 3D session page.

### The DNA methyltransferase 3B (DNMT3B) case study

**Fig. 6:**
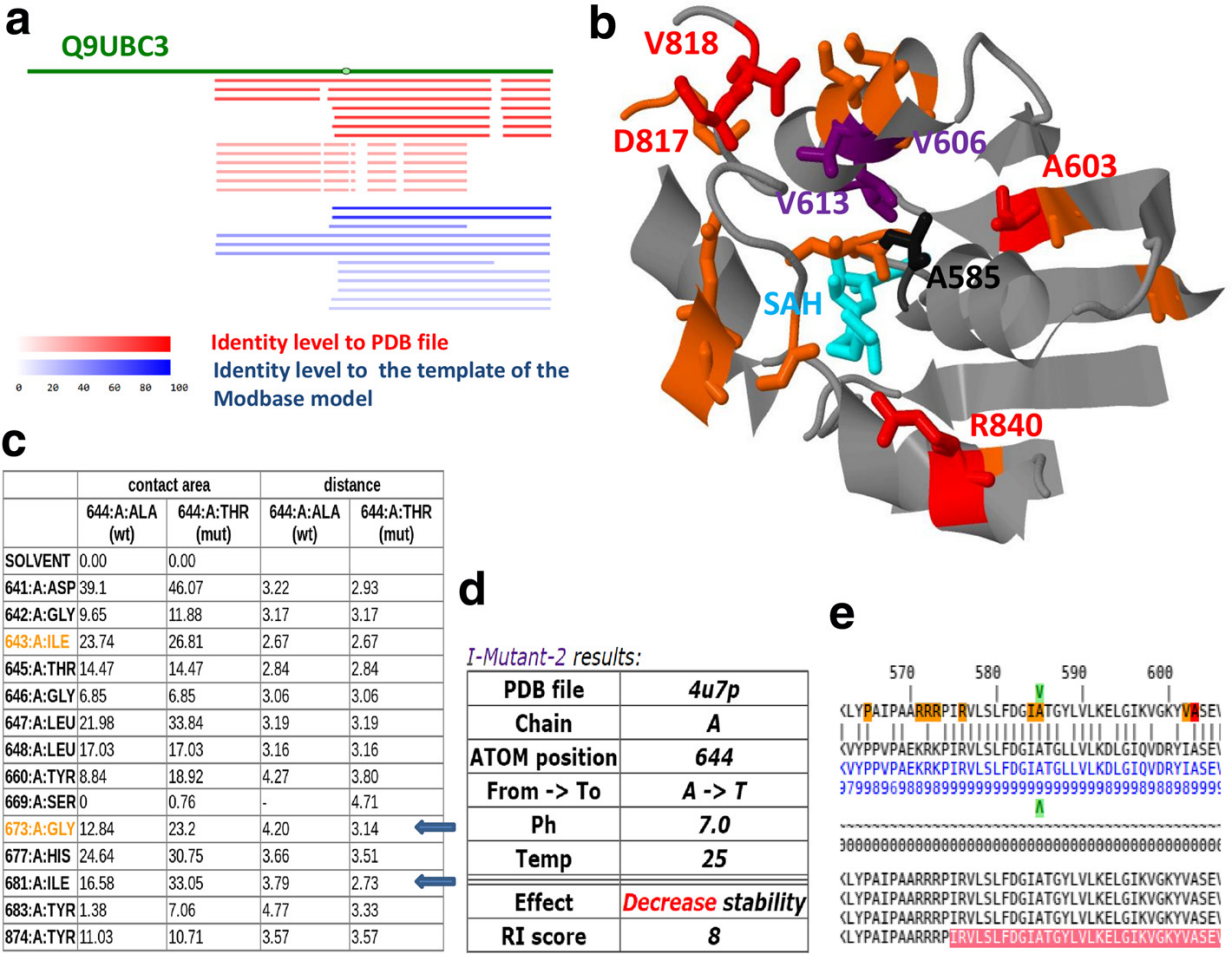
Example of G23D variant analysis in the catalytic domain of methyl-transferase (DNMT3B). Variants in this protein and specifically in position A585 are known to be related to the ICF syndrome. **a** DNMT3B has no resolved structures in the PDB, but by using G23D the user can rapidly detect structures of the close homolog DNMT3A (*red*) and several theoretical models which span the variant (*blue*). The color gradient indicates the sequence similarity to the PDB protein or to the template protein used for the homology modeling. **b** Analysis based on the structure of the catalytic domain of DNMT3A which shares 70 % identity with DNMT3B, suggests that the residue in position 585 (*shown in black*) is completely buried in the protein core and is located on the interface between a helix and a loop. It is positioned close to known pathogenic residues in both ClinVar (*red colors*) which are automatically being displayed in G23D and HGMD (here manually added in purple). Many other positions appear in Cosmic (*yellow*). The methyl donor (SAH), whose binding pocket is likely affected by mutations in position 585, appears in magenta. **c** From the sequence diagram, located in the bottom of the JSmol structure session it can be deduced that the region is very conserved (conservation score of 9) and that it is part of the known Prosite methyltransferase motif (PS51679; in pink). **d** Contact analysis shows that position 585 has direct contact with some of the known disease related positions shown in panel B, and that the minimal distance of the mutated residue causes steric clashes with two neighbors (*marked in arrows*). **e** Stability analysis of the A585T variant (equivalent to position 644 in PDB 4u7p ATOM section) using I-Mutant-2, directly accessed from G23D, suggests decreased stability of the mutant structure

DNMT3B is a gene responsible for *de novo* cytosine-5-methyltranferase (m^5^C) in the human genome. Familial mutations in this gene were reported to be the cause of autosomal recessive Immunodeficiency Centromeric instability and facial anomalies (ICF) syndrome [33]. Fig. 6 demonstrates how G23D can assist in meaningful structural analysis of a variant in this gene. This example will serve as an illustrative case study as well as a short tutorial for the utility of G23D.

A patient at two years of age was admitted to our hospital due to severe failure to thrive (FTT), immunodeficiency and diarrhea. She was the first born child of consanguineous parents from a Palestinian descent. In order to determine the genetic cause for her syndrome we performed whole exome sequencing (WES) of DNA extracted from her peripheral blood. The analysis revealed a strong candidate variant in chromosome 20, position 31387128 (hg19) from G to A, lying inside the genomic region of DNMT3B (c.1753G > A). Providing the genomic coordinates in the input page of G23D, instantly revealed that there are no PDB structure of DNMT3B. There are, however, several structures of the closely related DNMT3A (70 % identity in the catalytic domain) which cover the C-terminal part of the protein (Fig. 6a). The input variant resides in the catalytic domain located in the C-terminal part. There are also several homology models of the DNMT3B catalytic domain in ModBase. According to isoform Q9UBC3, the amino acid position affected by the change is Ala585 and the substituted amino acid is Thr (codon GCG to ACG). Note that if genomic position is provided, the protein position might differ for other isoforms.

By selecting the PDB hit bar of PDB file 4u7p [34], a detailed alignment between the query protein and the sequence in the PDB file (of DNMT3A) is presented. Position 585 is coded by nucleotides which appear to overlap cancer variants in Cosmic, as indicated by the yellow background. In fact, this position is also known to be an HGMD [35] variant, suggesting clinical importance. Several additional residues in the close vicinity are reported in cosmic and ClinVar databases. By clicking on the JSmol link, a 3D session appears, showing the structure (Fig. 6b). The input position 585 is depicted in black and the database variants are depicted in colors according to the G23D scheme. The structure of the threonine mutation residue can be seen in the 3D session by checking the “mut” button in the control panel. The list of contacts, available by a button click from the 3D session (Fig. 6c) reveals clashes with two adjacent residues (marked by arrows). Such clashes suggest significantly decreased stability of the mutant and/or changes in the local backbone conformation. Indeed prediction of I-Mutant2.0, directly accessed from the structural session, suggests a decreased stability of the mutant (Fig. 6d). Interestingly, the variant reported by Wijmenga et al. [36] in the same protein position also includes a larger mutated side chain (Val). Val has a similar shape like Thr, and is also expected to give rise to changes in the backbone conformation.

Moreover, many known clinical relevant variants are located in close proximity to position 585. The quantitative contact analysis (Fig. 6c), indeed suggests that position 585 (which appears as position 644 in the ATOM records of PDB 4u7p) has direct contact with other known pathogenic variants such as the residue 622 (681 in the PDB ATOM records). The region which spans position 585 is highly conserved as indicated by the “9” scores in the HSSP conservation profile (Fig. 6e). The sequence profile indicates that this region is part of a known motif of methyltransferases (SAM_MT_C5; Prosite ID: PS51679).

There is a consensus among eight different function prediction tools which are included in the dbNSFP database [15] that A585T is a deleterious change, as can be deduced by a link located in the upper part of the G23D hits’ page. DNMT3B variants described in the literature seem to disrupt the function of the enzyme by several distinct mechanisms, including changing protein stability, altering DNA binding affinity, affecting oligomerization with other methyltransferase family members and affecting binding affinity of the SAH methyl donor cofactor [37]. The variants in position 585 appear to belong to the last group. Ala 585 is located close to the SAH but does not directly contact it. Its side chain points to the opposite direction of the cofactor (shown in magenta in Fig. 6a) and is completely buried (Fig. 6c). Upon mutation, backbone changes are inevitable in order to accommodate the larger side chain. These changes are likely to affect the conformation of the nearby loop which directly interacts with the cofactor.

## Conclusions

Structural configuration is the basis for understanding molecular stability of molecules and interactions between molecules. It is also fundamental for understanding the molecular mechanisms which drive certain variants to be pathogenic. Due to the limited availability of structural data and technical difficulties in usage, structural information is often overlooked during functional interpretation of variants.

We believe that G23D will help to narrow this gap, and will allow more researchers to include protein structural aspects in their studies of human variants.

## Abbreviations

HTS: High throughput sequencing
PDB: Protein data bank
WES: Whole exome sequencing.
